# Effect of glycerol addition time on the cryopreserved Korean native brindle cattle (Chikso) sperm quality

**DOI:** 10.1590/1984-3143-AR2021-0058

**Published:** 2022-03-11

**Authors:** Lei Ma, Dae-Hyun Kim, Eun-Ju Jung, Woo-Jin Lee, Ju-Mi Hwang, Jeong-Won Bae, Dae-Jin Jung, Jun Koo Yi, Sang Moo Lee, Jae Jung Ha, Woo-Sung Kwon

**Affiliations:** 1 Department of Animal Science and Biotechnology, Kyungpook National University, Sangju, Gyeongsangbuk-do, Republic of Korea; 2 Gyeongbuk Livestock Research Institute, Yeongju Gyeongsangbuk-do, Republic of Korea; 3 Department of Animal Biotechnology, Kyungpook National University, Sangju, Gyeongsangbuk-do, Republic of Korea

**Keywords:** cryopreservation, glycerol adding time, Korean native brindle cattle (Chikso), Sperm functions

## Abstract

Although cryopreservation is an efficient method for maintaining the biological and genetic resources of sperm, the sperm damage during the cryopreservation process cannot be ignored. It should be possible to obtain the most effective cryopreservation performance by accurately grasping the effects of various factors on the cryopreservation of sperm. The previous study demonstrated that a suitable standard protocol for cryopreservation of Korean native brindled cattle (Chikso) does not exist, based on the methods for semen cryopreservation of Chikso differ in each research center. The most obvious difference between most of protocols is the addition of glycerol before and after cooling during the Chikso cryopreserved semen process. Therefore we focused on the effects of glycerol addition time on the quality of cryopreserved Chikso sperm. In the present study, 27 individual Chikso samples were collected by transrectal massage and divided into two parts: the “cryopreservation method A” group (adding glycerol before cooling) and the “cryopreservation method B” group (adding glycerol after cooling). Meanwhile, the values of various sperm parameters were derived from each group, including sperm motility, kinematics, capacitation status, cell viability, and intracellular ATP levels, which we used to compare and evaluate sperm function. The results of this study indicated that during the semen cryopreservation process of the Chikso, the addition of glycerol after cooling yielded superior results in a variety of sperm parameters, such as sperm motility, progressive motility, rapid motility, VCL, VSL, VAP, ALH, capacitation status, viability, and intracellular ATP level after freezing and thawing. Our study is suggested that the glycerol addition time during the cryopreservation process for Chikso should be considered. In addition, our results may be provided reference to develop suitable the cryopreservation procedure of the Chikso sperm.

## Introduction

Cryopreservation is a simple, economical, and efficient method for preserving the biological and genetic resources of sperm (Yánez-Ortiz et al, 2021). At present, it has been used in many fields and has become an essential means to treat human infertility, livestock reproduction, and biodiversity conservation of biological resources (Yánez-Ortiz et al, 2021; Aliakbari et al., 2021). However, sperm damage caused by cryopreservation cannot be ignored, such as cold shock, mitochondrial damage, acrosome damage, and others (Chaudhari et al., 2015; Yoon et al., 2015). Therefore, to ensure the maximum motility of frozen-thawed sperm in the process of sperm cryopreservation, it is critical to limit sperm potential damage, which requires careful consideration of various factors that may affect sperm motility in the cryopreservation process (Storey et al., 1998). The addition of cryoprotectant at the appropriate stage in sperm cryopreservation can effectively avoid or reduce the cryoinjury and cold damage of sperm to improve the motility of frozen-thawed sperm (Storey et al., 1998). In 1949, the researchers discovered the cryopreservation effect of glycerol on sperm (Polge et al., 1949). Since then, glycerol is often chosen to be used as a cryoprotectant and mammalian sperm from many species were successfully cryopreserved. In the cryopreservation of sperm, glycerol is the earliest and most widely-used antifreeze and used in the cryopreservation of sperm of almost all species till now is still a good cryoprotectant for cryopreservation of bovine sperm (Bhat et al., 2020; Gororo et al., 2020).

Currently, neither the cryopreservation method nor the cryoprotectant substance is suitable for the cryopreservation of sperm of all species. This situation is mainly due to the fact that sperm characteristics vary substantially between species and even between individuals, and react differently to freezing (Rosato and Iaffaldano, 2013). Therefore, it is necessary to study the cryopreservation process of sperm for different species and compare the cryopreservation effects of various kinds or concentrations of cryoprotectants on sperm. In the whole cryopreservation process, the type of cryoprotectant, its concentration, temperature, time of addition, the addition and removal methods, among others, affect the protective effect of sperm cryopreservation and these are issues that need to be considered in sperm cryopreservation (Bilodeau et al., 2001). Only by understanding the influence of these factors on sperm cryopreservation can cryoprotectants be used accurately to obtain the most effective cryopreservation performance.

Up to now, optimal procedure to crypreserve Chikso sperm is not been established. Therefore, the comprehensive management of the Chikso cryopreserved semen has not been implemented yet. Our previous study demonstrated that a suitable standard protocol for cryopreservation of Chikso does not exist, the significantly difference of various sperm parameters were observed in cryopreserved Chikso sperm using methods used for applying cryopreservation of other bulls (Ma et al., 2021). The most obvious difference between most of protocols is the addition of glycerol before and after cooling during the Chikso cryopreserved semen process. It has been reported that post-thaw motility of bison sperm was not observed significant effects by glycerol addition time (Vilela et al., 2017; Arif et al., 2020). However, other colleage have been showed that sperm motility was altered by glycerol addition time in freezing-thawing Mediterranean buffalo (B.bubalis) sperm (Fabbrocini et al., 2000). The different results suggest that the appropriate timing of glycerol addition can be different Depending on the species.

At present, no specific cryopreservation method has been established for the Chikso. Therefore, it is needed to develop a suitable technique for cryopreservation of Chikso. To this end, the present study was designed to investigate the effects of glycerol addition timing on the quality of cryopreserved Chikso sperm. Firstly, Chikso sperm were cryopreserved by two different cryopreservation methods based on the addition times of glycerol. Then, we measured various sperm parameters, including sperm motility, kinematics, cell viability, and intracellular ATP levels, from the sperm of the two groups. We used these variables from each group to compare and evaluate sperm function to reference the cryopreservation procedure of the sperm of the Chikso.

## Materials and methods

### Ethical statement

We performed all animal procedures following the guidelines for the ethical treatment of animals, and the study was approved by the Institutional Animal Care and Use Committee of Kyungpook National University (KNU 2017-141).

### Sample collection

The semen sample were collected from randomly selected 27 Chikso bulls (more than 2-year old) at the National Livestock Research Institute in Gyeongsangbuk-do. Before we sampled the semen, we removed the persistent feces from the bovine rectum. The operator inserted the hand into the rectum of the specimen about 25 cm, gently massaged the seminal vesicle gland to make the seminal vesicle secretions flow out from the foreskin, and then put the index finger between the two inflated parts of the vas deferens. Then, the operator put the middle finger and ring finger on the outside of one inflated part, and the thumb is placed on the outside of the other inflated part to massage it. During the massage, the fingers slid back and forth accompanied by some gentle pressure. This repeated massage caused the bull semen to flow out, allowing the assistant to insert the semen into the semen collection cup. Then, the assistant massages the S-shaped curved part of the penis of the bull to make it protrude beyond its prepuce, allowing the semen to be sampled and reducing the bacterial contamination (Palmer et al., 2004).

The samples were obtained from 27 individual Chikso samples and divided into two parts: the “cryopreservation method A” group (MA) and “cryopreservation method B” group (MB) based on the addition time of 12% glycerol Tris-egg yolk buffer.

### Cryopreservation of sperm

**Figure 1:**
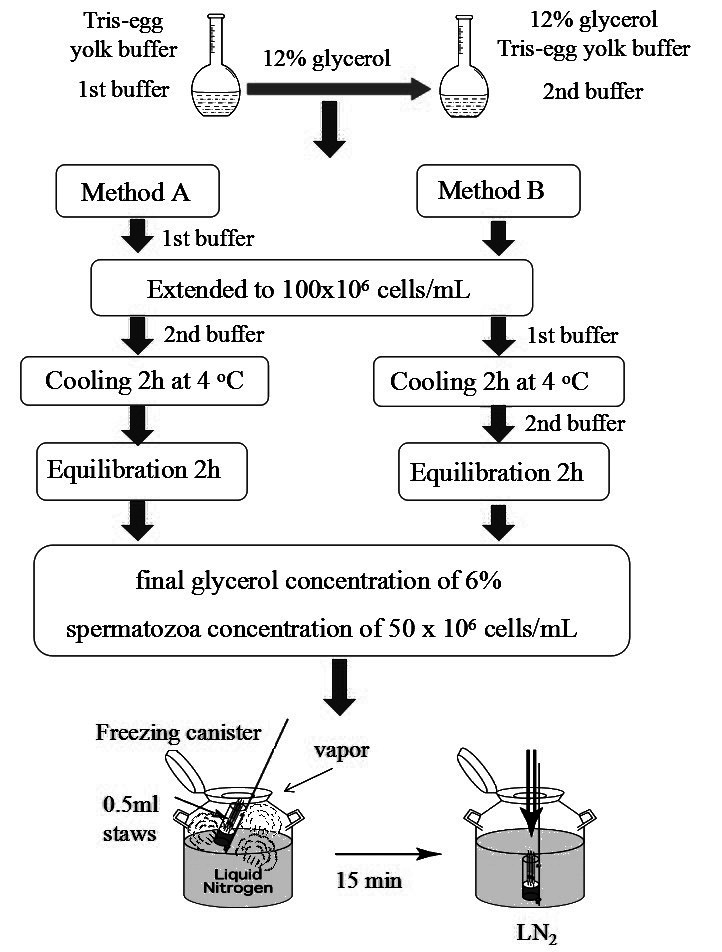
The process of sperm cryopreservation.

We prepared two kinds of buffers to extend the semen samples. The first buffer contained Tris-egg yolk buffer (TYB; 250 mM Tris, 88.5 mM citric acid, 68.8 mM glucose, 1.0g/L streptomycin, 0.57 g/L penicillin, and 20% egg yolk). The second buffer contained 12% glycerol Tris-egg yolk buffer (TYB; 250 mM Tris, 88.5 mM citric acid, 68.8 mM glucose, 1.0g/L streptomycin, 0.57g/L penicillin, 20% egg yolk, and 12% glycerol). We extended the semen samples from both groups to 100 × 10^6^ cells/ml using the first buffer, which contained no cryoprotectant at RT (Figure 1).

For the MA group: the semen samples were extended to 100 x 10^6^ cells/mL using the first buffer which containing no cryoprotectant at RT. The semen samples were extended again to 50 x 10^6^ cells/mL using the second buffer, resulting in a final glycerol concentration of 6%, and a final sperm concentration of 50 x 10^6^ cells/mL. Then cooled the samples at 4°C for 4 h (Figure1).

For the MB group: the semen samples were extended to 100 x 10^6^ cells/mL using the first buffer which containing no cryoprotectant at RT. First cooled the samples at 4°C for 2 h. An equal volume of the second buffer with 12% glycerol was added to extend the samples, resulting in a final glycerol concentration of 6%, and a final sperm concentration of 50 x 10^6^cells/mL then cooled again at 4°C for 2 h (Figure 1).

Equilibrated samples from two groups were packaged into 0.5-mL straws and frozen in liquid nitrogen vapor 2.5 cm above the liquid nitrogen for 15 min, then plunged into liquid nitrogen for storage (Figure 1). The cryopreservation methods for the present study were modified based on previously described (Yoon et al., 2015, 2016).

### Sample preparation

We carried out the sperm preparation with routine percoll washing as previously described (Lee et al., 2009; Brugnon et al., 2013). We thawed the frozen sperm samples for 20 s in a 39 °C water bath. To remove extender debris, seminal plasma, cryoprotectant and diluent added during sperm cryopreservation, the samples were centrifuged for 20 minutes at 400 × g with a discontinuous Percoll density gradient from 1 ml 90% to 1 ml 45% Percoll. We discarded the top layer of the suspension after centrifugation. We washed the sperm pellet at 400 × g with 1 ml Tyrode’s albumin lactate pyruvate (TALP) medium for 5 min. Then, we extracted the supernatant and applied 500 L of TALP medium. The TALP medium contained 1.0 mM sodium pyruvate, 100 mM NaCl, 0.4 mM MgCl·6H_2_O, 3.1 mM KCl, 0.3 mM Na_2_HPO_4_·12H_2_O, 2.0 mM CaCl·2H_2_O, 25 mM NaHCO_3_, 21.6 mM sodium lactate, and 0.6% bovine serum albumin.

### Computer-assisted sperm analysis

We used a computer-assisted sperm analysis program (CASA) (FSA2016, Medical supply, Seoul, Korea) with a CMOS image sensor and a 2,048 × 1,536 (300 megapixels), 60 frame camera (Medical supply, Seoul, Korea) and an OLYMPUS BX43 phase-contrast microscope (Olympus, Tokyo, Japan) with a 10 × objective phase-contrast mode to evaluate sperm motility and motion kinematics. In a preheated (37 ºC) Makler counting chamber, we assessed a 10 μl smear of the sample (Sefi-Medical Instruments, Haifa, Israel). The movement of at least 250 sperm cells was recorded for each sample from random fields (>5). We analyzed the obtained images to determine the following: 1) sperm motility (%), 2) progressive sperm motility (%), 3) rapid sperm motility (%), 4) medium sperm motility (%), 5) slow sperm motility (%), 6) curvilinear velocity (VCL, µm/s), 7) straight line velocity (VSL, µm/s), 8) average path velocity (VAP, µm/s), 9) beat cross frequency (BCF, Hz), and 10) amplitude of lateral head displacement (ALH, µm).

### Assessment of capacitation status by Hoechst 33258/chlortetracycline fluorescence

We used the Hoechst 33258 (H33258)/chlortetracycline fluorescence (CTC) dual staining method (combined Hoechst 33258/chlortetracycline fluorescence assessment) to assess capacitation status. We centrifuged the samples at 400 × g for 5 min. After removing the majority of the supernatants, we added 135 μl of DPBS and 15 μl of H33258 solution (10 g H33258/ml DPBS) to the remaining samples. After 3 min of room temperature incubation (RT), we added 250 μl of 2% percent (w/v) polyvinylpyrrolidone in DPBS to the mixture to remove excess dye by layering. Then, we washed the sample by centrifugation at 400 × g for 5 min. We resuspended the pellet in 50 μl of CTC solution after the supernatant was extracted completely. (750 mM CTC in 5 μl buffer: 20 mM Tris, 5 mM cysteine, 130 mM NaCl, pH 7.4) and 50 μl DPBS. Then, we smeared the samples onto slides in 10 μl increments. For each sample, we counted at least 400 sperm on each slide. We used filters for H33258 and CTC, an OLYMPUS BX43 with epifluorescence illumination, and ultraviolet BP 340–380/LP 425 and BP 450–490/LP 515 excitation/emission (Olympus, Tokyo, Japan). Finally, we observed the following capacitation patterns: live acrosome-reacted sperm (AR pattern, no fluorescence over the head, or green fluorescence only in the post-acrosomal region); live capacitated sperm (B pattern, the acrosomal region showed bright green fluorescence, and the region showed dark post-acrosomal); sperm that is not incapacitated and is alive (F pattern, bright green fluorescence that is evenly spread across the sperm head); and dead sperm (D pattern, blue fluorescence distributed uniformly over the sperm head).

### Detection of intracellular ATP level

We quantified the intracellular ATP generation using an ATP Assay Kit (ab83355, Abcam, Cambridge, UK) using the manufacturer’s instructions. Briefly, the samples (1 x 10^6^ cells) were centrifuged at 10,000 × g for 5 min, and removed the majority of the supernatants. Then the sample was washed by 1 × PBS (4°C), and 50 μl of sperm was placed into 96-well plates. Before the experiment, we primed the ATP reaction mix and equilibrated it to room temperature. Then, we added the reaction mixture in equal volume to each well and incubated them at RT for 30 min, shielded from light. Finally, we measured luminescence with a microplate reader at OD 570 nm (Gemini Em; Molecular Devices Corporation, Sunnyvale, CA, USA). We used SoftMax Pro 7 software (Molecular Devices Corporation, Sunnyvale, CA, USA) to analyze it.

### Assessment of sperm viability

We used the Abcam Cell Assay Kit (ab112118, Abcam, Cambridge, UK) to monitor cell viability. It uses a proprietary water-soluble dye that changes its absorption spectra upon cellular reduction. The absorption ratio change is directly proportional to the number of living cells. Before experiments, we thawed the assay solution and preheated it to 37 °C. Then, we placed 100 μl of sperm and 20 μl of assay solution in a 96-well plate. The sample was then incubated for 2 h in the incubator at 37 °C in 5% CO_2_ protected from light. We measured the cell viability and monitored the absorbance OD 570 nm and 605 nm (Gemini Em; Molecular Devices Corporation, Sunnyvale, CA, USA). We evaluated the cell viability with the ratio of OD 570 nm to OD 605 nm (SoftMax Pro 7; Molecular Devices Corporation, Sunnyvale, CA, USA).

### Statistical analysis

We classified the samples into two groups by glycerol addition time. We obtained numerical values in each group from different sperm parameters (such as motility, motion kinematics, capacitation status, viability, and ATP level) from the 27 individual samples. Data were analyzed with SPSS (Version 25.0, IBM, Armonk, NY, USA). We used the Student’s t-test of variance to compare the values of the two groups, considering all the sperm parameters we assessed. We represent all data as means ± standard error of mean (SEM). We considered the P-values < 0.05 as statistically significant.

## Results

### Sperm motion parameters

**Figure 2:**
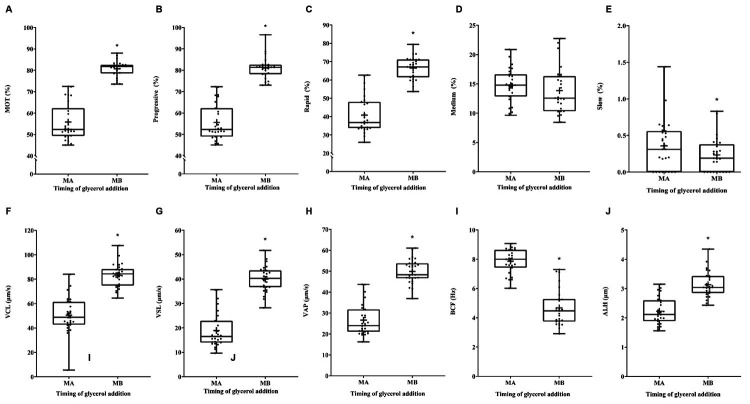
The effect of glycerol addition time on Korean Native Brindled Cattle (Chikso) cryopreserved sperm motility and motion kinematics. (**A**) MOT = Sperm motility (%); (**B**) Progressive = Progressive sperm motility (%); (**C**) Rapid = Rapid sperm motility (%); (**D**) Medium = Medium sperm motility (%); (**E**) Slow = Slow sperm motility (%); (**F**) VCL = Curvilinear velocity (μm/s); (**G**) VSL = Straight line velocity (μm/s); (**H**) VAP = Average path velocity (μm/s); (**I**) BCF = Beat cross frequency (Hz); (**J**) ALH = Mean amplitude of head lateral displacement (μm). MA = Method A. MB = Method B. (*P < 0.05, n = 27).

The CASA program was used to monitor sperm motility and motion kinematics of the two groups of samples. The results showed that a variety of motion parameters, including sperm motility, progressive motility, rapid motility, VCL, VSL, VAP, and ALH were significantly higher in the MB group compared to the MA group of sperm. While slow motility, and BCF were significantly higher in the MA group compared to the MB group of sperm. However, there were no differences between samples from the two groups concerning medium motility (P < 0.05, Figure 2, Supplementary Material Table 1).

### Sperm capacitation status

**Figure 3:**
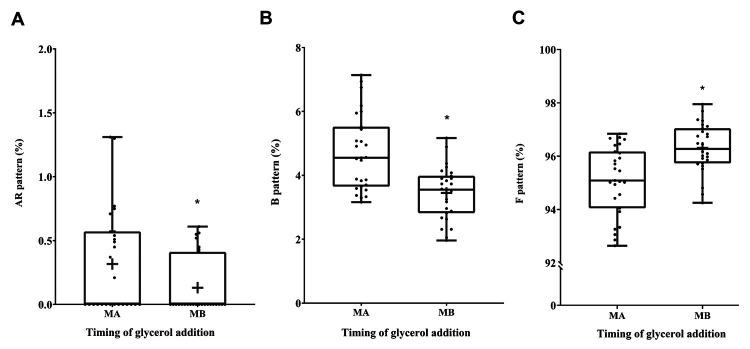
The effect of glycerol addition time on cryopreserved Korea Native Brindled Cattle (Chikso) sperm capacitation status. (**A**) Patterns of live acrosome reacted (AR pattern); (**B**) Patterns of live capacitated (B pattern); (**C**) Patterns of live non-capacitated (F pattern); MA = Method A. MB = Method B. (*P < 0.05, n = 27).

CTC/H33258 dual staining was used to determine sperm capacitation status. The results showed that the capacitated B pattern was significantly lower in the MB group (3.45 ± 0.82%) compared to the MA group (4.71 ± 1.20%) of sperm. However, the non-capacitated F pattern was significantly higher in the MB group (96.31 ± 0.91%) compared to the MA group (95.10 ± 1.29%) of sperm. In the case of the AR pattern, the acrosome-reacted sperm was significantly lower in the MB group (0.19 ± 0.26%) compared to the MA group (0.24 ± 0.41%) (P < 0.05, Figure3).

### ATP level in sperm

**Figure 4:**
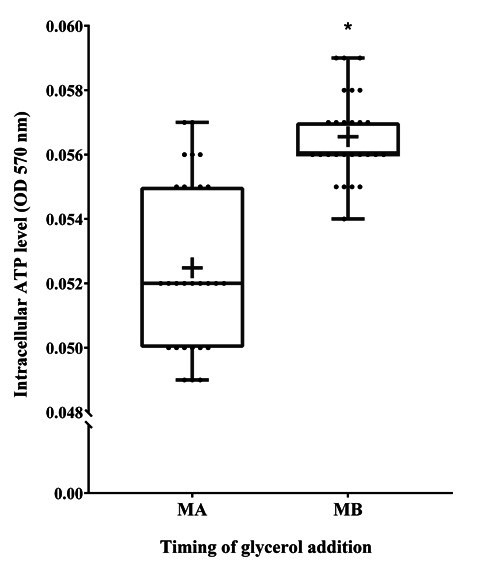
The effect of glycerol addition time on cryopreserved Korean Native Brindled Cattle (Chikso) sperm intracellular ATP level. MA = Method A. MB = Method B. (*P < 0.05, n = 27).

Quantitative ATP measurements were conducted using an ATP assay kit. The results showed that the intracellular ATP level of the samples was significantly higher in the MB group (0.057 ± 0.00026) compared to the MA group (0.052 ± 0.00051%) sperm (P < 0.05, Figure 4).

### Cell viability

**Figure 5:**
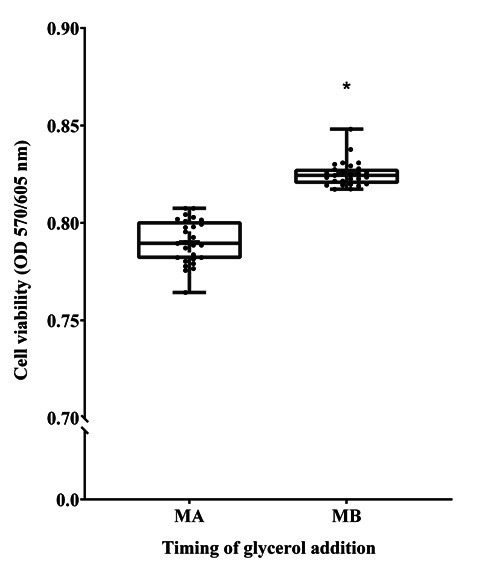
The effect of glycerol addition time on cryopreserved Korean Native Brindled Cattle (Chikso) sperm cell viability. MA = Method A. MB = Method B. (*P < 0.05, n = 27).

Monitoring cell viability is one of the essential tasks for the comparative assessment of sperm function. We evaluated the calorimetric detection as a measure of cell viability. The results showed that the cell viability of the samples in the MB group (0.826 ± 0.0019) was significantly higher compared to the MA group (0.789 ± 0.0034) sperm (P < 0.05, Figure 5).

## Discussion

Cryopreservation can cause low temperatures that damage sperm acrosome, plasma membrane, mitochondria and sperm function (Khan et al., 2021). During the process of cryopreservation, cryoprotant addition diminish the chemical and physical dysfunction caused by freezing and thawing (Jang et al., 2017). However, at the same time, it will affect sperm function (Macías García et al., 2012). Therefore, the addition of cryoprotant in a appropriate way plays a vital role in sperm survival and fertilization ability (Watson, 2000).

In this study, we measured various sperm parameters, including sperm motility, kinematics, cell viability, and intracellular ATP levels, from the sperm of the two groups. We evaluated the effect of two cryopreservation method with different glycerol adding time on cryopreserved semen quality of Chikso.

The results showed that a variety of motion parameters, including sperm motility, progressive motility, rapid motility, VCL, VSL, VAP, and ALH were significantly lower in the MA group compared to the MB group of sperm (P < 0.05, Figure 2, Supplementary Material Table 1). However, there were no differences between samples from the two groups concerning medium motility (P < 0.05, Figure 2, Supplementary Material Table 1). As glycerol is a small molecule compound that can penetrate cell membranes to enter cells. The addition of glycerol helps to maintain cell volume and ion interaction during cryopreservation (Holt, 2000). It has been reported that the sperm can be stored for 24 hours at 5°C without losing motility and fertility (Martins et al., 2009). Additionally, the cooling process during the sperm cryopreservation has no significant effect on sperm motility (Yoon et al., 2015). The addition of glycerol before cooling will only cause the change of osmotic pressure (Rosato and laffaldano, 2013). This procedure can change the permeability of the membrane by replacing the water in the cell, causing a change in sperm volume, leading to the destruction of sperm surface proteins, thus the loss of motility and reduced fertilization ability (Rosato and laffaldano, 2013). Kinematic and morphological changes are essential prerequisites for fertilization. Moreover glycerol addition before cooling causes oxidative damage to sperm membrane phospholipids, resulting in the loss of motility (Watson, 2000; Yoon et al., 2016). Consistent with these findings, significantly lower sperm motility, significantly higher slow motility, and significantly higher BCF in MA group were observed in current study. (P < 0.05, Figure 2, Supplementary Material Table 1).

Cold shock is directly related to the cooling process of cryopreservation, which alters the physical characteristics and functioning of cell membranes (White, 1993; Miller et al., 2004). In the process of cooling at 4°C, the fluidity of the membrane, the tolerance to changes in osmotic pressure, and the intracellular potassium concentration are all disturbed, thereby enhancing the uptake of Ca^2+^ in sperm cells (Watson, 2000; Melville et al., 2012; Yoon et al., 2016).

Furthermore, the addition of cryoprotectant before cryopreservation only exacerbates the osmotic pressure changes, membrane instability, sperm volume changes, calcium influx, and protein denaturation (Rosato and Iaffaldano, 2013). Our results showed that the acrosome-reacted sperm was significantly higher in the MA group compared to the MB group. The capacitated pattern was significantly higher in the MA group compared to the MB group of sperm. In the case of the non-capacitated pattern was significantly lower in the MA group compared to the MB group of sperm (P < 0.05, Figure 3). This might be attributed to the addition of glycerol before cooling that induces calcium influx hence triggers the acrosome reaction and capacitation. Subsequently, various sperm parameters that are critically related to the fertilization ability of sperm are altered, such as viability, motility and capacitation status (Liu and Foote, 1998; Watson, 2000; Yoon et al., 2016). Correspondingly, the cell viability of the samples in the MA group was significantly lower compared to the MB group sperm (P < 0.05, Figure 5).

Oxidative stress causes alterations in sperm metabolism and function (Liu and Foote, 1998; Sorrenti et al., 2014; Yoon et al et al., 2016). The energy (ATP) used for sperm motility is mainly caused by mitochondrial respiration (Kastelic and Thundathil, 2008). Therefore, there is a close relationship between exercise and mitochondrial activity. Consistent with these findings, the intracellular ATP level of the samples was significantly lower in the MA group compared to the MB group sperm (P < 0.05, Figure 4).

The freeze–thaw step of cryopreservation is related to the formation of ice crystals, usually accompanied by the change of osmotic pressure (Yoon et al., 2015). After cooling, adding glycerol to the cryopreservation solution can effectively protect sperm from cryopreservation injury while minimizing damage to the cells. Its action mechanism mainly includes the following two aspects: outside the cell, the permeable antifreeze is easy to hydrate, increasing the viscosity of the solution, increasing the osmotic pressure of the solution, and leading to cell dehydration, thus reducing the formation of ice crystals in the cell. The permeable cryoprotectant enters the cells to partially replace the water and reduce the cellular water content, thus reducing the formation of intracellular ice crystals and preventing rapid changes in cell volume caused by changes in osmotic pressure (Rajan and Matsumura, 2018). Dehydration, during freezing and resuscitation, thereby protects the mitochondria and other organs of the cells (Baust et al., 2009). Correspondingly, a variety of sperm parameters, such as sperm motility, progressive motility, rapid motility, VCL, VSL, VAP, ALH, viability, intracellular ATP level of sperm in the MB group was significantly higher than the MA group (P < 0.05, Figures 2, 4, 5, Supplementary Material Table 1). Therefore, during the semen cryopreservation process of the Chikso, the quality indexes of sperm after freezing and thawing were better with the addition of glycerol after cooling.

## Conclusion

The results of this study indicated that during the semen cryopreservation process of the Chikso, the addition of glycerol after cooling yielded superior results in a variety of sperm parameters. Therefore, we suggest that the glycerol addition time should be considered during the cryopreservation process for Chikso sperm. In addition, our results may be provided reference to the cryopreservation procedure of the Chikso sperm.

## Supplementary Material

Supplementary material accompanies this paper.

Supplementary Table 1 The effect of glycerol addition time on the motility and motion kinematics of cryopreserved sperm of the Korean native brindle cattle (Chikso)

This material is available as part of the online article from https://www.scielo.br/j/ar
