# Supplementary material for: Effect of glycerol addition time on the cryopreserved Korean native brindle cattle (Chikso) sperm quality
Source: Anim Reprod. 2022 Mar 11;19(1):e20210058. doi: 10.1590/1984-3143-AR2021-0058 (PMC9000158; doi:10.1590/1984-3143-AR2021-0058)
Supplement: Supplementary Table 1 [file 1984-3143-ar-19-1-e20210058-suppl01.pdf]

Supplementary Table 1. The effect of glycerol addition time on the motility and motion kinematics of cryopreserved sperm of the Korean native brindle cattle (Chikso).

Parameters	Values		Max.		Min.	
	MA	MB	MA	MB	MA	MB
MOT (%)	55.79 ± 8.47	80.76 ± 3.68*	72.46	88.08	45.05	73.54
Progressive (%)	55.58 ± 8.48	81.04 ± 4.92*	72.32	96.59	45.05	73.02
Rapid (%)	40.84 ± 10.12	66.52 ± 6.06*	62.67	79.41	26	53.69
Medium (%)	14.74 ± 2.90	13.85 ± 3.97	20.88	22.74	9.65	8.43
Slow (%)	0.36 ± 0.35	0.23 ± 0.23*	1.44	0.83	0	0
VCL (%)	50.51 ± 14.88	83.21 ± 9.58*	84.04	107.6	5.46	64.52
VSL (%)	18.96 ± 7.32	39.96 ± 5.36*	35.65	51.77	9.65	28.22
VAP (%)	26.64 ± 7.60	49.89 ± 5.79*	43.72	61.05	16.25	36.93
BCF (Hz)	7.87 ± 0.82	4.68 ± 1.25*	9.07	7.30	6.02	2.91
ALH (µm)	2.23 ± 0.46	3.13 ± 0.44*	3.15	4.35	1.56	2.43

MOT = Sperm motility (%); Progressive = Progressive sperm motility (%); Rapid = Rapid sperm motility (%); Medium = Medium sperm motility (%); Slow = Slow sperm motility (%);VCL = Curvilinear velocity (µm/s); VSL = Straight line velocity (µm/s); VAP = Average path velocity (µm/s); BCF = Beat cross frequency (Hz); ALH = Mean amplitude of head lateral displacement (µm). MA = Before cooling. MB = After cooling. Data are presented as mean ± SEM. (*$P < 0.05$, n = 27)
